# Attenuation of Tick-Borne Encephalitis Virus Using Large-Scale Random Codon Re-encoding

**DOI:** 10.1371/journal.ppat.1004738

**Published:** 2015-03-03

**Authors:** Lauriane de Fabritus, Antoine Nougairède, Fabien Aubry, Ernest A Gould, Xavier de Lamballerie

**Affiliations:** 1 Aix Marseille Université, IRD French Institute of Research for Development, EHESP French School of Public Health, EPV UMR_D 190 “Emergence des Pathologies Virales”, Marseille, France; 2 Institut Hospitalo-Universitaire Méditerranée Infection, Marseille, France; Integrated Research Facility at Fort Detrick, UNITED STATES

## Abstract

Large-scale codon re-encoding (*i*.*e*. introduction of a large number of synonymous mutations) is a novel method of generating attenuated viruses. Here, it was applied to the pathogenic flavivirus, tick-borne encephalitis virus (TBEV) which causes febrile illness and encephalitis in humans in forested regions of Europe and Asia. Using an infectious clone of the Oshima 5–10 strain ("wild-type virus"), a cassette of 1.4kb located in the NS5 coding region, was modified by randomly introducing 273 synonymous mutations ("re-encoded virus"). Whilst the *in cellulo* replicative fitness of the re-encoded virus was only slightly reduced, the re-encoded virus displayed an attenuated phenotype in a laboratory mouse model of non-lethal encephalitis. Following intra-peritoneal inoculation of either 2.10^5^ or 2.10^6^ TCID50 of virus, the frequency of viraemia, neurovirulence (measured using weight loss and appearance of symptoms) and neuroinvasiveness (detection of virus in the brain) were significantly decreased when compared with the wild-type virus. Mice infected by wild-type or re-encoded viruses produced comparable amounts of neutralising antibodies and results of challenge experiments demonstrated that mice previously infected with the re-encoded virus were protected against subsequent infection by the wild-type virus. This constitutes evidence that a mammalian species can be protected against infection by a virulent wild-type positive-stranded RNA virus following immunisation with a derived randomly re-encoded strain. Our results demonstrate that random codon re-encoding is potentially a simple and effective method of generating live-attenuated vaccine candidates against pathogenic flaviviruses.

## Author Summary

The arbovirus Tick-borne encephalitis virus (TBEV; genus *Flavivirus*) is transmitted by ticks of the *Ixodes* genus. TBEV causes febrile illness and encephalitis in humans in forested regions of Europe and Asia. The incidence of TBE is increasing across Central and Eastern European countries despite the availability of several licensed inactivated vaccines and appropriate vaccination programmes. Large-scale codon re-encoding, a recently developed attenuation method that modifies viral RNA nucleotide composition of large coding regions without alteration of the encoded proteins, has been successfully applied to a variety of RNA viruses. In contrast with previous empirical methods of generating live attenuated vaccines, large-scale codon re-encoding facilitates rapid generation of vaccine candidates using reverse genetics methods, by direct control of the attenuation phenotype. Additional benefits include reduced costs and induction of long-term immunity. Here, we have applied the large-scale codon re-encoding method to the TBEV to demonstrate the principle of developing a live attenuated virus vaccine which protects mice against subsequent infection with the wild type virulent virus. This study therefore illustrates that codon re-encoding is potentially an easily derived and effective method of producing live attenuated vaccine candidates against positive-stranded RNA viruses.

## Introduction

The genus *Flavivirus* (family *Flaviviridae*) includes important human pathogens such as yellow fever virus (YFV), dengue virus (DENV), Japanese encephalitis virus (JEV), West Nile virus and tick-borne encephalitis virus (TBEV). Flaviviruses are enveloped, single-stranded positive-sense RNA viruses with virions, close to 50nm in diameter, and a viral genome of *ca*. 11 kb which includes a unique open reading frame (ORF) encoding structural (C-prM-E) and non-structural proteins (NS1–2A-2B-3–4A-4B-5) [1,2]. Some mosquito-borne flaviviruses also harbour sequences that induce a proportion of translating ribosomes to shift-1 nt and continue translating in the new reading frame to produce a ‘transframe’ fusion protein [3,4]. Most flaviviruses are arboviruses and are therefore maintained in nature by circulating between haematophagous arthropod vectors and vertebrate hosts. Arthropod-borne flaviviruses are sub-divided into two major groups: the tick-borne and mosquito-borne flaviviruses (TBFVs and MBFVs respectively) [1,2,5]. TBFVs include a heterogeneous group called seabird tick-borne flavivirus group (S-TBFV) [6] and the mammalian tick-borne flavivirus group (M-TBFV), with all known pathogenic TBFVs causing febrile illness, encephalitis and/or haemorrhagic fever in humans. In the latter group, TBEVs are recognised in 25 European and 7 Asian countries and transmitted by *Ixodes* species ticks [7]. The TBEVs are subdivided into three sub-types, namely Siberian, Western European and Far Eastern viruses [8,9], the latter being responsible for the most severe forms of central nervous system (CNS) disorders associated with high fatality rates (5–20%) [10]. Despite the availability of several licensed inactivated vaccines and vaccination programmes [1,11], the incidence of TBEV infections is increasing across much of Central and Eastern European countries, currently with an estimated 9,000 cases per year [12,13,14].

Live attenuated vaccines provide effective and affordable protection against major flaviviral infections. One dose of the widely used 17D YF vaccine used since 1937, provides long-lasting immunity [15]. The live attenuated JE vaccine (strain SA-14–14–2) has been successfully used in China with over 100 million children vaccinated [16]. Attenuated strains of virus have been obtained in the past using empirical methods such as serial passage of wild-type (WT) viruses in cell cultures and/or chicken embryos. Whilst several hundred million yellow fever vaccine doses have been administered, and proven to be safe and highly efficacious [16,17], the attenuation mechanism is associated with a number of non-synonymous mutations (31 in the case of the 17D-204 YF vaccine strain when compared with the WT Asibi virus). These modifications can occasionally generate new biological properties, *e*.*g*., a neurovirulent phenotype for YF 17D in contrast to the viscerotropic phenotype of WT yellow fever viruses. New approaches to resolve such problems are required.

Large-scale codon re-encoding is a recently developed method with which to attenuate virus by introducing a large number of slightly deleterious synonymous mutations into the protein coding region of the viral genomic RNA without alteration of the encoded proteins. Genomic re-encoding has been successfully applied to a variety of RNA viruses: poliovirus, influenza A virus, chikungunya virus, human respiratory syncytial virus, human immunodeficiency virus, Japanese encephalitis virus and porcine reproductive and respiratory syndrome virus [18,19,20,21,22,23,24,25,26,27]. In each of these examples, re-encoding modulated virus fitness thus generating potential vaccine candidates with antigenically indistinguishable proteins, therefore alleviating the generation of novel and therefore undesirable biological properties. To date, most published studies have utilised specific re-encoding approaches, including codon deoptimisation, codon-pair deoptimisation or increase of CpG/UpA dinucleotide frequency. The choice of these methods is based on the assumption that global modification of the viral genome induces attenuation because synonymous sites have been shaped by genome-wide mutational processes during virus evolution [28]. However, a random re-encoding approach, previously applied *in cellulo* to the chikungunya virus, also produced an attenuated phenotype without modifying the global properties of the viral genome, thus underlining the role of local constraints that also shape synonymous sites such as secondary structures or interactions between viral RNA and capsid proteins [27,29,30,31]. Therefore, whilst the efficacy of re-encoding methods for attenuating RNA viruses has been widely demonstrated, the precise—and presumably multiple- mechanisms and their respective contributions to attenuation remain to be analysed in more detail. *In cellulo* experiments showed, for poliovirus, chikungunya virus and bacterial virus T7, that the phenotype of the re-encoded viruses was stable, and that the evolutionary response to re-encoding was compensatory in nature, with very few reversion mutations [22,27,32].

In the present study, we have applied the random large-scale codon re-encoding method to the TBEV Oshima 5–10 strain, isolated in Japan in 1995 [33]. This strain belongs to the Far Eastern TBEV subtype which is characteristically highly neurovirulent for mice [34,35]. Our studies demonstrate that this re-encoded TBEV Oshima 5–10 strain exhibited an attenuated phenotype *in vivo* and induced robust protective immunity in mice subsequently infected with the WT virus.

## Results

**Fig 1:**
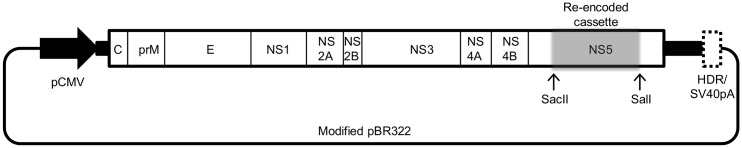
Schematic representation of the cloning vector pTBEV-32.11 ic (WT_IC). Coding (white rectangles) and non-coding (black rectangles) regions which represent the complete genome of the TBEV WT_IC were flanked in 5′ and 3′ by the pCMV and the HDR/SV40pA, and inserted into a modified pBR322 plasmid. The re-encoded cassette is represented by a grey rectangle flanked by the restriction sites SacII and SalI.

An infectious clone derived from the wild-type Oshima 5–10 TBEV strain was constructed using reverse genetics methods (see the Materials and Methods section for more details). This infectious clone was recovered from cell cultures and designated “WT_IC virus”. In addition, a re-encoded “NS5_Reenc_IC virus” was derived from WT_IC virus by substituting a cassette of approximately 1.4kb, in the corresponding NS5 coding region, with the re-encoded counterpart (Fig. 1). We have chosen to introduce mutations into the NS5 coding region because the first re-encoded cassette introduced into the Chikungunya virus, as described in our previous work [27], was located in the nsP4 coding region which also encoded the viral RNA dependent RNA polymerase. This enabled comparisons, *in cellulo* and *in vivo*, of the biological properties of the WT and re-encoded viruses.

### *In silico* analysis

A total of 273 synonymous mutations, located between positions 8,619 and 10,019 (with reference to the complete genome sequence, GenBank accession number KF623542), was introduced in the specified NS5 coding region using a random distribution algorithm with restrictions [27] (Table 1). The codon usage (measured using the effective number of codons; eNC) and G+C% of the NS5_Reenc_IC virus was slightly modified compared with that of the WT_IC virus: 53.96 *vs* 55.46 and 54.3% *vs* 53.8%, respectively (Table 1). When compared with 85 TBEV and 56 other TBFV complete ORF sequences retrieved from GenBank, the eNC value of the NS5_Reenc_IC virus was higher than the maximum eNC value of TBEV sequences but fell within the extreme values of all available TBFV sequences. On the other hand, the G+C% value of NS5_Reenc_IC virus fell within the extreme values of TBEV sequences.

**Table 1:** Genetic characteristics of the complete coding regions of WT_IC and NS5_Reenc_IC viruses, and of 85 other tick-borne encephalitis viruses (TBEV) and 56 other tick-borne flaviviruses (TBFV).

Virus	Localisation of the re-encoded cassette	Re-encoded cassette size	Number of synonymous mutations	eNC	G+C%
**WT_IC**	-	-	-	53.96	54.3
**NS5_Reenc_IC**	NS5 Position 8,619–10,019	1,412 nt	273	55.46	53.8
**85 TBEV sequences [min; max]**	-	-	-	[53.33; 55.24]	[53.8; 55.3]
**56 other TBFV sequences [min; max]**	-	-	-	[52.35; 57.29]	[52.1; 55.3]

Codon usage bias was evaluated using the effective number of codons (eNC) which gives a value ranging from 20 (only one codon used for each amino-acid) to 61 (random codon usage for each amino-acid). eNC and G+C% values were calculated using the CodonW v1.3 software.

### *In cellulo* replicative fitness

WT_IC and NS5_Reenc_IC viruses were recovered following transfection of the corresponding infectious clones in BHK21 cells. These two viruses were then passaged once on BHK21 cells and their replicative fitness was subsequently studied.

#### Growth kinetics

**Fig 2:**
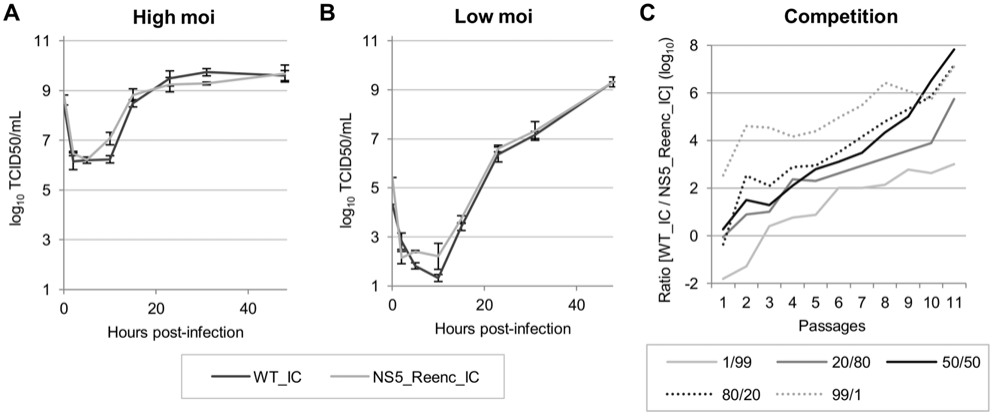
*In cellulo* replicative fitness of WT_IC and NS5_Reenc_IC viruses in BHK21 cells. Results of growth kinetics curves performed at high (200) (**A**) or low (0.5) (**B**) initial moi (Results are expressed as mean/standard deviation values). Results of competition experiments (**C**) performed using five initial TCID50 ratios (WT_IC/NS5_Reenc_IC: 1/99, 20/80, 50/50, 80/20, 99/1). Two qRT-PCR assays, specific for WT_IC or NS5_Reenc_IC virus, enabled to monitor the evolution of the proportion of each virus along 11 passages.

Growth kinetics in BHK21 cells of both viruses were compared at high (200) and low (0.5) multiplicity of infection (moi). Infectious cell supernatant was harvested at 2, 5, 10, 15, 23, 31 and 48 hours post-infection. The infectious titre in each sample was estimated using a TCID50 assay. Regardless of the initial moi, growth kinetics of WT_IC and NS5_Reenc_IC viruses were essentially similar (Fig. 2A-B).

#### *In cellulo* competition experiments

Competition experiments can provide sensitive comparisons of the replicative fitness of two viruses [27]. Using the method described previously for chikungunya virus [27], five initial TCID50 ratios (WT_IC/NS5_Reenc_IC virus: 1/99, 20/80, 50/50, 80/20, 99/1) and a combined moi of 0.5 were used to infect BHK21 cells. Infectious cell supernatants were then passaged 11 times in BHK21 cells. Two specific quantitative RT-PCR techniques (each specifically detecting one of the competing viruses) were used to determine the proportion of each viral genome in cell supernatants at each passage (expressed as log_10_ in Fig. 2C). These competition experiments showed that WT_IC virus was fitter than NS5_Reenc_IC virus when the initial TCID50 ratios were compared. Indeed, by the 11^th^ passage, the WT_IC/NS5_Reenc_IC ratio had increased by at least 10,000 fold.

### *In vivo* experiments

**Fig 3:**
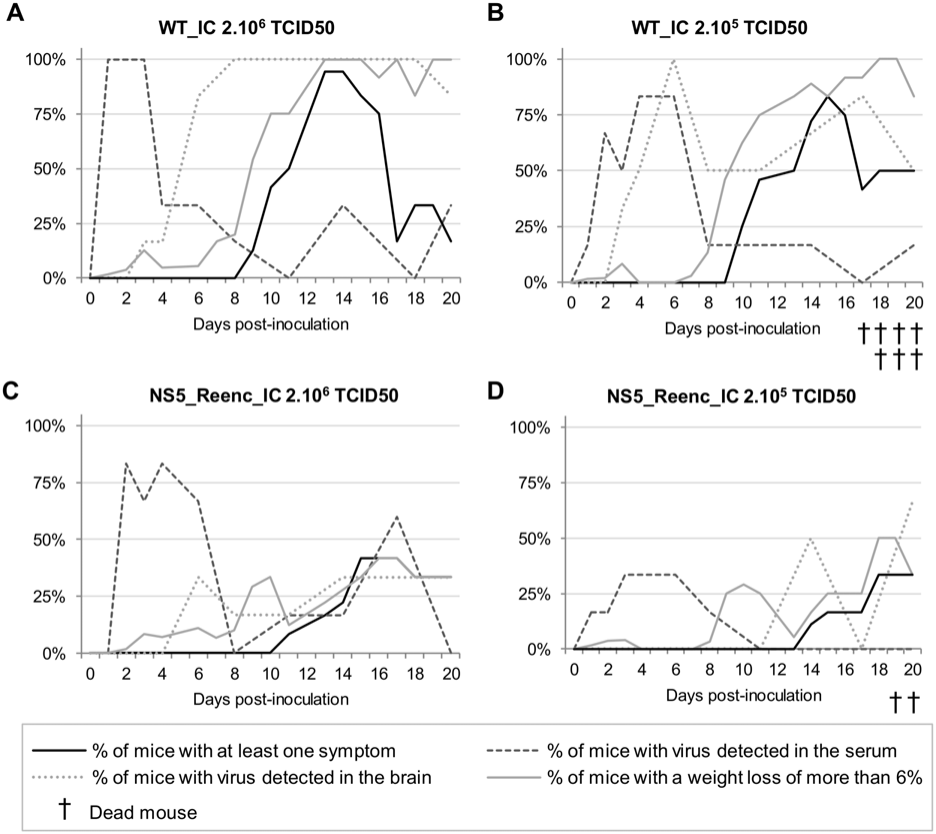
Time distribution of the proportion of mice with at least one symptom, with a weight loss of more than 6% and with virus detected in the serum or in the brain by qRT-PCR. Mice were inoculated with 2.10^6^ TCID50 of WT_IC virus (**A**), 2.10^5^ TCID50 of WT_IC virus (**B**), 2.10^6^ TCID50 of NS5_Reenc_IC virus (**C**) or 2.10^5^ TCID50 of NS5_Reenc_IC virus (**D**).

The laboratory mouse model has been the primary global choice with which to study the CNS pathology induced by TBEVs. Pathologic changes in mouse brains as well as clinical signs are similar of those observed in humans [35,36,37]. In agreement with previous findings, virus recovered from infectious clones displayed lower mortality than the original cell culture derived Oshima 5–10 strain which induces high mortality in mice following intra-peritoneal inoculation [34]. Therefore, mortality was not used as a comparative criterion: we only observed late and low mortality rates when the mice were inoculated with the lower dose of TBEV (Fig. 3). It should also be noted that TBEVS are known to infect small rodents chronically/persistently as suggested by field studies [38,39].

Five-week-old C57Bl/6J female mice were inoculated intra-peritoneally with 200μL containing 2.10^5^ TCID50 [low dose] or 2.10^6^ TCID50 [high dose] of virus. Clinical monitoring included *(i)* the clinical manifestation of the disease (shivering, humpback, dirty eyes, weak paws, hemiplegia or tetraplegia) and *(ii)* the body weight curve (a weight loss of more than 6% of the initial weight was chosen as a disease recognition criterion, as described in S1 Fig in S1 Text). Periodically, groups of mice were sacrificed to conduct virological investigations. A close relationship between viral load values determined using either quantitative RT-PCR (qRT-PCR) or the TCID50 method was observed. Consequently, the virological follow-up of sera and brains was performed using qRT-PCR (S2A and S2B Fig in S1 Text). Sera were also used for viral serological analysis by ELISA and virus neutralisation assays.

#### Comparative study of pathogenicity

Four groups of 60 mice were inoculated with two doses (2.10^5^[low-dose] or 2.10^6^[high-dose] TCID50) of either WT_IC virus or NS5_Reenc_IC virus. A control group of 16 mice was inoculated with PBS. Mice were weighed daily and monitored for the appearance of symptoms during the 20 day period of observation. Six mice per group were periodically euthanized to perform virus detection in sera and brains (at day 1, 2, 3, 4, 6, 8, 11, 14, 17 and 20 post-inoculation (p.i.)).

**Fig 4:**
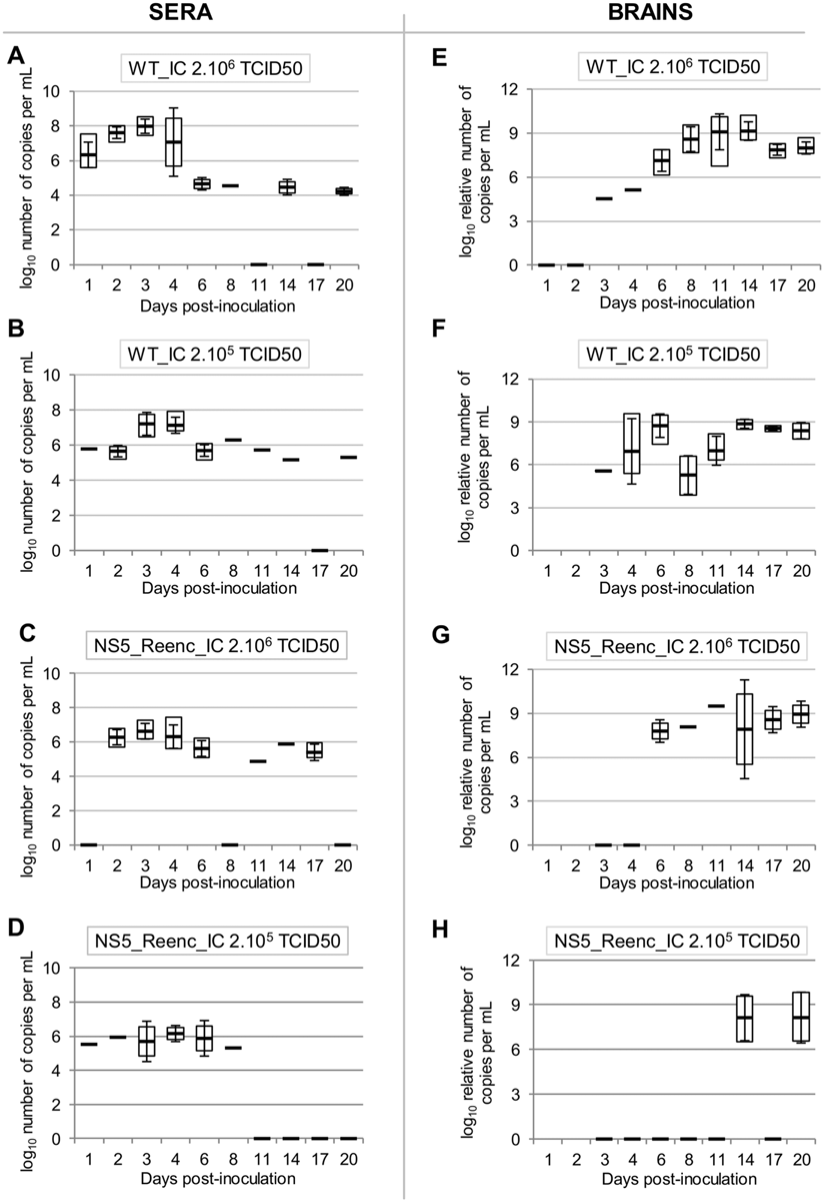
Viral RNA yields in sera (A-D) and brains (E-H). The virus and the dose used are indicated in each graphic. Viral RNA yields are expressed as log_10_ number of copies per mL (sera) or log_10_ relative number of copies per mL (brains) (values were normalized using the housekeeping gene HMBS as seen in the Materials and Methods section). White squares represent minimal and maximal values. Black lines and error bars represent mean values +/- standard deviation. A black line with a value of zero means that all samples were negative.

**Fig 5:**
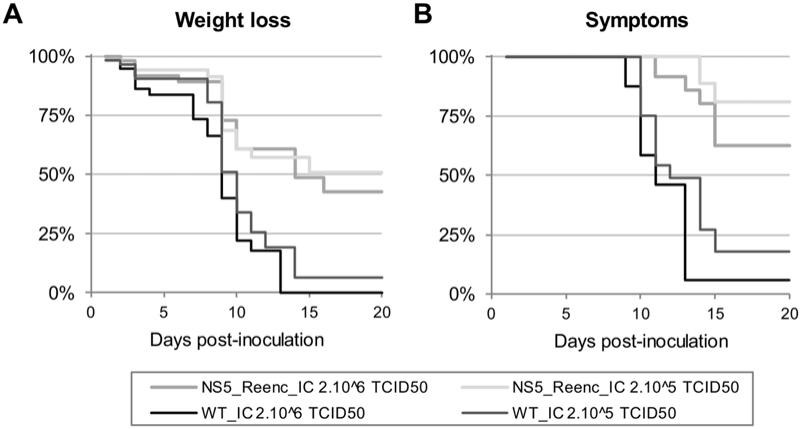
Kaplan-Meier survival analysis using as criteria, a weight loss of more than 6% (A) and appearance of at least one symptom (B).

The proportion of viraemic animals was not significantly different between the high-dose and low-dose WT groups (Fig. 3A-3B), or between the high-dose re-encoded (Fig. 3C) and WT groups (Fig. 3A-3B). By contrast, it was significantly lower in the low-dose re-encoded group (Fig. 3D) compared with either the high-dose re-encoded (Fig. 3C) (*p* = 0.012, Fisher exact test) or WT groups of mice (Fig. 3A and 3B) (*p*<0.001, Fisher exact test). In all groups, the highest proportion of viraemic animals was observed 3 to 7 days p.i. Viral RNA yields found in sera were heterogeneous and no significant difference was found in term of inoculation dose or strain of virus (WT_IC or NS5_Reenc_IC viruses) inoculated (Fig. 4A-4D). In the WT-infected mice the peak period for detection of virus in the brain (neuroinvasion) always followed the peak viraemic titres, and preceded the appearance of encephalitic symptoms (see Fig. 3A-3B). In the groups infected by re-encoded virus, the kinetics of these parameters was atypical. Both weight loss and encephalitic symptoms were significantly less frequent than in the WT groups, regardless of the inoculation dose (Mandel-Cox’s Logrank test: *p*<0.0001 and *p* = 0.004 for mice infected with low and high doses, respectively; see Kaplan-Meier analysis in Fig. 5). The proportion of animals with virus in the brain (neuroinvasion) was significantly higher in WT-infected mice compared with re-encoded virus-infected mice, regardless of the dose used (Fisher exact test: p<0.0001 for both low- and high-dose groups, respectively; see Fig. 3). Viral RNA yields found in brains were heterogeneous and no significant difference was found in term of inoculation dose or strain of virus (WT_IC or NS5_Reenc_IC viruses) inoculated (Fig. 4E-4H).

#### Serological investigations

**Fig 6:**
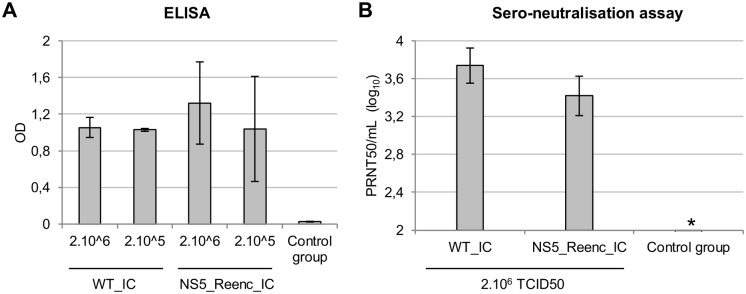
Results of TBEV serology at day 43 post-inoculation. ELISA tests **(A)** and Sero-neutralisation tests **(B)**. Results are expressed in the graphs as mean/standard deviation values for each group of mice. * all the sera of the control group tested were negative (all the samples were under the detection threshold of the method (2.25 PRNT50/mL)).

Five groups of 4 mice were inoculated with either a low- or high-dose of WT_IC virus or NS5_Reenc_IC virus. A control group of non-infected mice was included. Forty three days after infection, the serum of each mouse was tested for the presence of antibodies to TBEV (only 3 mice were tested for the WT_IC 2.10^6^ TCID50 group because one mouse died). TBEV-specific immunoglobulin G (IgG) antibodies were detected using a commercial diagnostic ELISA kit. High antibody titres were detected in mice infected by both the WT and the re-encoded viruses. Mice in the control group were negative (Student’s t-test; *p*<0.0001) (Fig. 6A). Sero-neutralisation tests were performed on the mouse sera representing mice inoculated with 2.10^6^ TCID50 of virus. Neutralising antibodies were detected in all mice, but not in mice from the control group (Fig. 6B).

#### Challenge experiments

Forty days after the inoculation of two groups of mice with the NS5_Reenc_IC virus and two groups of mice with the WT_IC virus (at both high and low doses for both viruses), 8 mice per group were challenged with 2.10^6^ TCID50 of WT_IC virus, and the protection was evaluated by determining for each group the proportion of mice *(i)* viraemic at day 2 post-challenge, *(ii)* with brains positive for the presence of TBEV at day 12 post-challenge and *(iii)* with a weight loss of more than 6% of the weight at days 11 and 12 post-challenge. Two control groups of mice were used: one group of 8 mice inoculated with PBS and then challenged with virus as described above (mock group), and one group of 4 mice inoculated and challenged with PBS (used to normalise the weight of the mice).

Protection was 100% effective in terms of viraemia, for challenged mice immunised with the NS5_Reenc_IC virus or the WT_IC virus (both doses). In contrast, all mice in the mock group were viraemic (0/4 versus 4/4; Fisher’s exact test; *p* = 0.03 for each dose). Amongst all the weighing realised at days 11 and 12 post-challenge (S4 Table in S1 Text), only one showed a weight loss of more than 6% amongst mice immunised with the NS5_Reenc_IC virus (one mouse initially vaccinated with 2.10^5^ TCID50 of NS5_Reenc_IC virus, at day 12 post-challenge, not at day 11 post-challenge). We found similar results with the challenged mice previously inoculated with the WT_IC virus: only one mouse inoculated with 2.10^5^ TCID50 of WT_IC virus was below the detection threshold, only at day 12 post-challenge. In contrast, 87.5% of the weighing realised with the mice of the mock group were under this threshold (Fisher’s exact test; *p*≤0.01 when compared separately for each initial inoculation dose).

Surprisingly, we detected viral RNA in the brains of 100% of the challenged mice previously inoculated with the WT_IC virus and 100% of mice of the mock group. Viral RNA yields found in brains ranged from 10^3.7^ to 10^6.8^ relative number of copies per mL and no significant difference was found between the different groups of mice. We also detected viral RNA in the brains of 25% (1/4) and 75% (3/4) respectively of the challenged mice previously vaccinated with high and low doses of NS5_Reenc_IC virus. To determine whether or not the detected virus was the result of persistence in the brain, we quantified viral RNA yields with our two specific quantitative RT-PCR assays that were used previously in competition experiments (see above). For the challenged mice previously infected by the NS5_Reenc_IC virus, we found that the viral RNA present in the brain at day 12 post-challenge was exclusively the NS5_Reenc_IC virus but we failed to isolate the virus from these brain samples. Therefore, we obtained a protection of 100% in terms of neuroinvasion by the virus used for the protection experiments (0/4 versus 4/4; Fisher’s exact test; *p* = 0.03). However, for the mice vaccinated with the WT_IC virus, because the same virus was used to infect and challenge these mice, we could not draw valid conclusions in this instance.

#### *In vivo* competition experiments

**Fig 7:**
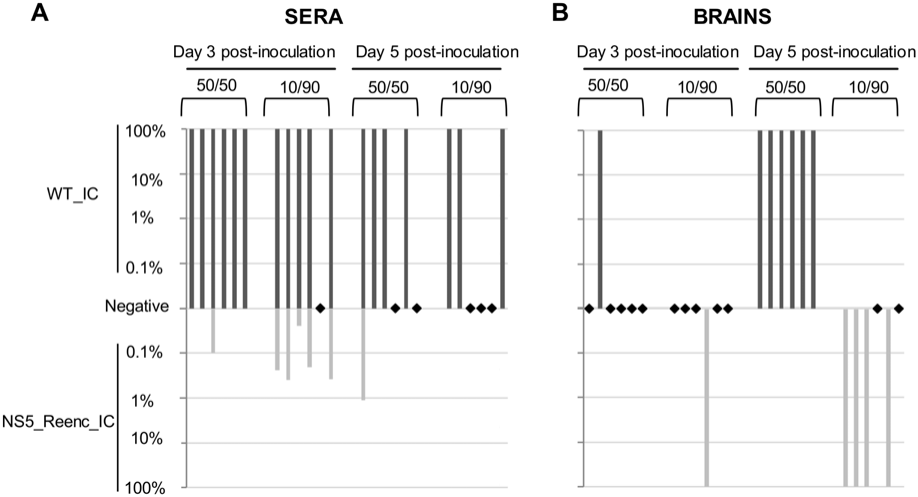
Results of *in vivo* competition experiments. Competitions were performed using two initial PFU ratios (WT_IC/NS5_Reenc_IC virus: 50/50 or 10/90). Two qRT-PCR assays, specific for WT_IC or NS5_Reenc_IC virus enabled to quantify the proportion of each virus in the viral population in sera (**A**) and in brains (**B**). Dark and light grey rectangles represent the percentage of WT_IC and NS5_Reenc_IC virus in each sample (percentages are represented using a log_10_ scale). Black diamonds represent samples for which qRT-PCR were negative for WT_IC and NS5_Reenc_IC detection.

*In cellulo* competition experiments had shown that WT_IC virus had a higher replicative fitness than NS5_Reenc_IC virus (see above). We therefore conducted *in vivo* competition experiments to determine whether or not the loss of replicative fitness due to re-encoding had an impact on viraemia and viral yields in the brain. Two initial TCID50 ratios (WT_IC/NS5_Reenc_IC virus: 10/90, 50/50—total dose: 2.10^6^ TCID50) were used to inoculate two groups of six mice. At days 3 and 5 p.i., one group of mice per initial TCID50 ratio was sacrificed and the relative proportions of each viral genome in sera and brains was estimated using RT-PCR as reported in the earlier example of *in cellulo* competition assays (expressed as log_10_ in Fig. 7).

In sera, regardless of the composition of the initial inoculum, the WT virus was the most represented in the viral population at days 3 and 5 p.i. (Fig. 7A). However, the results in brains were different: in all samples, only one of the 2 viruses was identified. The composition of the initial mixed inocula determined which virus was identified: the WT virus was recovered when the WT_IC/NS5_Reenc_IC ratio was 50/50 but the NS5_Reenc_IC virus was recovered when this ratio was 10/90 (Fig. 7B). The significance of these results is discussed further below.

## Discussion

We have evaluated *in cellulo* and *in vivo* the effect of genomic large-scale random re-encoding on TBEV), a pathogenic TBFV that causes febrile illness and encephalitis in humans. Encephalitic flavivirus infections provoke CNS pathology which can be correlated with the observed morbidity and mortality: viruses replicating in the CNS induce direct neuronal damage causing severe CNS dysfunction often involving long-term neurological sequelae in non-fatal cases [36,40,41]. In addition, it has been demonstrated that host immune response is a critical determinant of clinical outcome [36,42,43,44,45].

Here, an infectious clone of a neurovirulent strain of TBEV (Far Eastern subtype) was used to perform *in vivo* studies in a mouse model that faithfully mirrors many aspects of the infection in humans. Our experiments showed that decreased replicative fitness (as determined by *in cellulo* competition assays and *in vivo* viraemia measurement) was associated with reduced neuroinvasiveness as previously described [46]. *In vivo* competition experiments shed further light on the mechanisms of pathogenesis: when a 50/50 (WT/Reenc) TCID50 initial ratio was used, as expected, the wild-type virus rapidly out-paced the re-encoded virus in blood and was the only virus detected in mouse brains. However, when a 10/90 (WT/Reenc) TCID50 initial ratio was used, the re-encoded virus was the only one detected in brain, suggesting an early and selective neuroinvasion process: only the majority virus present in blood during the first hours of the viraemia invades the central nervous system. Of course, more experiments are needed to confirm these findings and the potential role of viral interferences have to be assessed [47,48].

In this model, TBEV infection is frequently associated with asymptomatic persistence of the virus in mouse brain as previously described [38,39] and confirmed by our experiments (we detected viral RNA of the re-encoded virus but failed to isolate the virus). Previous reports of virus reactivation, years after the initial infection, suggest that this specific phenomenon might also occur in humans [49].

The main objectives of this study were *(i)* to analyse the effect of genome random re-encoding on the fitness and clinical phenotype of a virulent TBEV strain and *(ii)* to perform a complete set of *in vivo* experiments including immunisation with a re-encoded virus and follow-up challenge experiments with an infectious wild-type virus. It has previously been demonstrated that large-scale re-encoding generates attenuated viruses and the studies support the proposal that relative degree of attenuation or replicative fitness can be regulated by modulating the number of introduced synonymous mutations [18,19,20,21,22,23,24,25,26,27]. Indeed, a re-encoded strain of influenza A virus that displayed limited fitness *in cellulo* was highly attenuated when tested in a mouse model and showed potential for use as a vaccine candidate [25]. Similarly, re-encoding a 1.4 kb region of the CHIKV genome by introducing 300 random synonymous mutations was associated with only limited *in cellulo* attenuation [27]. Therefore, we hypothesised that appropriate re-encoding of a TBEV strain might result in limited fitness reduction *in cellulo* and thus provide a relevant candidate to study the relative degree of *in vivo* attenuation. Accordingly, we introduced 273 random synonymous mutations in the NS5 gene of a neurovirulent strain of TBEV (Oshima, Far Eastern subtype), with limited modification of G+C content or codon bias of the genome. This re-encoding protocol produced a virus variant that displayed no fitness difference when growth kinetics were compared with WT TBEV in mammalian cell cultures. However, more sensitive *in cellulo* competition experiments revealed that the replicative fitness of the wild-type virus was indeed higher than that of the re-encoded virus. Moreover, *in vivo* experiments in immunocompetent mice fully validated our starting hypothesis: the re-encoded virus could reproduce efficiently in mice but in competition experiments the wild-type virus had a significantly higher fitness. *In vivo* experiments also revealed the attenuated characteristics of the re-encoded virus, namely reduced neurovirulence in terms of weight loss and appearance of neurological symptoms and reduced neuroinvasiveness (*i*.*e*. lower proportion of mice with virus in the brain). It can therefore be concluded that the re-encoding process, although restricted to the NS5 gene, decreased the pathogenicity and led to the production of an attenuated phenotype of the normally highly virulent strain of TBEV.

Concerning the mouse protection experiments, the results showed that, at 40 days post-infection, neutralising antibodies were produced by 100% of mice, *i*.*e*. infected by either the wild-type or the re-encoded virus. Moreover, no significant differences in antibody titres were observed between mice infected with either virus. Thus, mice “immunised” by the re-encoded virus were likely to have been protected against subsequent TBEV infection. This was verified at 40 days post-infection by challenging the “immunised” mice using a high dose of intraperitoneally administered wild-type virus. Protection was effective in terms of viraemia, neurovirulence and neuroinvasion. Likewise, inoculation of CD155 tg mice with re-encoded strains of poliovirus induced the production of neutralising antibodies and protects the mice against a subsequent challenge with a lethal dose of virus [24].

The results provide a robust proof of concept: large-scale random re-encoding can be used to produce *in vivo* attenuated strains of TBEV and infection of mice by re-encoded viruses can induce neutralising and protective immune responses against challenge with virulent homologous viruses. This represents evidence that a positive-stranded randomly re-encoded RNA virus could be developed and trialled as a potential vaccine to protect humans and/or animals against viral pathogens.

Many current virus vaccines were derived empirically and carry an inherent risk of vaccine-associated complications. Our findings open up new perspectives for the development of new-generation custom-designed re-encoded live-attenuated vaccines which are potentially safe, induce high levels of protective immunity and are relatively easy to produce.

The use of a highly neurovirulent strain of TBEV in our experimental model enabled us to identify the *in vivo* modification of the clinical picture provided by genome re-encoding. The results strongly suggest that re-encoding could be used in the future for attenuation of highly pathogenic viral species. However, it would be wiser and more practical in the specific case of TBEV, to develop a live attenuated re-encoded vaccine using a strain known to have a naturally lower association with neurovirulence and by inserting additional synonymous mutations in coding regions with a view to reducing the encephalitic potential of the virus and thus produce a potentially safer vaccine candidate. Whilst effective inactivated vaccines are available to prevent TBEV infections, the use of a live attenuated vaccine may have specific advantages, *e*.*g*. long-term protection and reduced costs [50].

## Materials and Methods

### Cells and animals

Baby hamster kidney BHK21 (BHK21) cells (ATCC, number CCL10) and mouse (L929) cells (ATCC, number CCL1) were grown at 37°C with 5% CO_2_ in Minimum Essential Medium with 7% fetal calf serum (Life Technologies) and 1% Penicillin/Streptomycin (5000U/mL and 5000μg/mL; Life Technologies). Five-week-old C57Bl/6J mice females were provided by Charles River laboratories.

### Ethics statement

Animal protocols were reviewed and approved by the ethics committee “Comité d’éthique en expérimentation animale de Marseille—C2EA—14” (protocol number 2504). All animal experiments were performed in compliance with French national guidelines and in accordance with the European legislation covering the use of animals for scientific purposes (Directive 210/63/EU).

### *In silico* re-encoding method

A cassette of 1,412 bp located in the NS5 coding region was randomly re-encoded as described previously for chikungunya virus [27]. Briefly, a computer programme was used to randomly attribute nucleotide codons based on their corresponding amino acid sequence: for example, the amino acid proline was randomly replaced by CCT, CCC, CCA or CCG. The number and the position of rare codons in primate genomes [51] (*i*.*e*. CGU, CGC, CGA, CGG, UCG, CCG, GCG, ACG), and unique restriction sites were conserved (S1 Note in S1 Text).

### Construction of TBEV infectious clones (ICs)

We modified a previously described IC of the Oshima 5–10 strain [34] (GenBank accession number of the parent virus: AB062063) by adding 9 synonymous mutations along the genome to increase the number of unique restriction sites, by replacing the SP6 promoter by a promoter CMV (pCMV) in 5′, by adding in 3′ of the complete viral genome the sequence of the hepatitis delta ribozyme followed by the simian virus 40 polyadenylation signal (HDR/SV40pA). The origin of replication was replaced by a modified pBR322. This IC was designated Cloning vector pTBEV-32.11 ic (GenBank accession number KF623542) and was considered as WT (Fig. 1). The re-encoded cassette (see above) was synthesized *de novo* by GenScript and inserted into the WT IC by digestion (SacII/SalI; New England Biolabs) (Fig. 1), gel purification of digestion products (Qiagen), ligation (T4 DNA ligase; Life Technologies) and transformation into electrocompetent STBL4 cells (Life Technologies). Before their transfection, both ICs were purified (0.22μm filtration) and their genome integrity was verified using a restriction map and complete sequencing.

### Sequence analysis

Complete open reading frames of TBEV (n = 85) and other TBFV (n = 56) were manually extracted from GenBank (S2 and S3 Tables in S1 Text). G+C% and effective number of codons (eNC) were calculated using Codon W v1.3 software [52,53].

### Recovery of infectious viruses and stock production

ICs were transfected into a 12.5cm^2^ culture flask containing sub-confluent BHK21 cells (FuGENE 6 transfection reagent; Roche). After incubation for 6 hours, cells were washed twice with Hank’s Balanced Salt Solution (HBSS, Life Technologies) and incubated until appearance of complete cytopathic effect (CPE). Cell supernatant medium was harvested, clarified by centrifugation and stored at -80°C. Each virus was then passaged in BHK21 cells at a calculated moi of 0.5 in a 175cm^2^ culture flask: after adsorption of the virus for 2 hours, the cells were washed twice (HBSS) and 50mL of medium was added and the flasks were incubated at 37°C for 72 hours. Cell supernatant media were harvested, clarified by centrifugation, aliquoted, stored at -80°C and used to perform *in cellulo* experiments. A similar experimental procedure was also carried out using L929 cells and the resulting cell supernatant medium was aliquoted, stored and used to perform the *in vivo* experiments. The integrity of the genome of all the viruses produced to perform *in cellulo* and *in vivo* experiments was verified using sequencing methods (Sanger methods).

### Virus replication kinetics

A calculated moi of 200 or 0.5 was used to infect a 25cm^2^ culture flask of confluent BHK21 cells. Cells were washed twice (HBSS) 30 minutes after the infection and 7mL of medium was added. 800μL of cell supernatants were sampled just before the washes and at 2, 5, 10, 15, 23, 31 and 48 hours post-infection. They were clarified by centrifugation, aliquoted and stored at −80°C. They were then analyzed using a TCID50 assay (see below).

### *In cellulo* competition experiments

As described previously for chikungunya virus WT_IC virus was competed with NS5_Reenc_IC virus [27]: five initial TCID50 ratios (WT_IC/NS5_Reenc_IC virus: 1/99, 20/80, 50/50, 80/20, 99/1) were used to infect a 25cm^2^ culture flask of confluent BHK21 cells at a calculated moi of 0.5. Cells were washed twice with HBSS and then incubated for 48h after addition of 7mL of medium. Recovered infectious cell supernatant was then sequentially passaged 10 times in the same manner with the clarified cell supernatant medium from the previous passage. At each passage, a calculated moi of 1 was used. Aliquots of cell supernatant from each passage were clarified by centrifugation and stored at -80°C. Viral RNA was extracted from clarified culture supernatant medium using the EZ1 Virus Mini Kit v2 on the EZ1 Biorobot (both from Qiagen). Using two specific quantitative real time RT-PCR assays targeting the re-encoded NS5 coding region (see the quantitative real time RT_PCR assays section for more details), the amount of viral RNA was assessed for each virus (WT_IC and NS5_Reenc_IC) and the ratio of the two values (WT_IC/NS5_Reenc_IC) was calculated.

### *In vivo* experiments

Five-weeks-old C57Bl/6J female mice were intra-peritoneally inoculated with 200μL containing 2.10^5^ TCID50 or 2.10^6^ TCID50 of virus. In some experiments (see details in the results section), a control group of mice was used (they were intra-peritoneally inoculated with 200μL of PBS). The clinical course of the viral infection was monitored by following (i) the clinical manifestation of the disease (shivering, humpback, dirty eyes, hemi- or tetra-paresia, hemiplegia or tetraplegia) and (ii) the weight of the mice. Weights were normalized with the average weight of mice of control group; the normalized weight was expressed as percentage of initial weight and calculated as follows: (% of initial weight: weight/weight at the day of the inoculation or challenge)–(mean of the % of the initial weight for control mice) +100. Brains and blood were sampled from sacrificed mice. Blood was collected by intracardiac puncture. After centrifugation, serum was aliquoted and stored at -80°C. Nucleic acid extraction using 50μL of serum previously inactivated with 50μL of AVL buffer (Qiagen) and spiked with 10μL of MS2 bacteriophage (internal control) was performed using the EZ1 Virus Mini Kit v2 on the EZ1 Biorobot (both from Qiagen). Brains were collected in 1mL of PBS with a tungsten bead and ground using a TissueLyser (Qiagen) for 3min at 30cycles/s. The brain suspensions were homogenized with NucleoSpin filters (Macherey-Nagel). The collected filtrate was then aliquoted and stored at -80°C. Virus TCID50 assays were performed using these filtrates. Nucleic acid extraction using 30μL of filtrate, 270μL of RLT buffer (Qiagen) and 10μL of MS2 bacteriophage (internal control) was performed using the EZ1 RNA Tissue Mini Kit on the EZ1 Biorobot (both from Qiagen).

### Virus isolation using brain samples

100μL of the filtrates collected from brain suspensions (see above) were used to inoculate a 12.5cm^2^ culture flask of confluent BHK21 cells containing 400μ of medium. After incubation for 2 hours, 2.5mL of medium was added and cells were incubated 5 days. A blind passage was then realised using a 25cm^2^ culture flask of confluent BHK21 cells. 2mL of clarified cell supernatant diluted 1:3 was used to inoculate the cells. After incubation for 2 hours, cells were washed once with HBSS and incubated 5 days. Virus replication was demonstrated using detection of viral genomes in cell supernatant using qRT-PCR assay and detection of cytopathic effect (CPE).

### Quantitative real-time RT-PCR assays

All quantitative real-time PCR (qRT-PCR) assays were performed with SuperScript III Platinium One-Step qRT-PCR kit. The mix content for a final volume of 25μL per sample, was as follows: a standard quantity of 2x of PCR Mastermix and Enzymes, both primers (final concentration: 0.4μM), probe (final concentration: 0.1μM) and 4μL of extracted nucleic acids. qRT-PCR were performed on CFX96 Real-Time System/C1000 Touch Thermal Cycler (Biorad) with the following conditions: 15min at 50°C, 2min at 95°C, then 45 times 15sec at 95°C and 40sec at 60°C, data collection occurring during this last step. Primers and probe sequences are detailed in S1 Table in S1 Text. All sera and brain samples from mice were spiked with MS2 bacteriophage (internal control) prior nucleic acid extraction and a MS2-specific qRT-PCR was performed to monitor the extraction, reverse transcription, and amplification steps as previously described [54]. A universal qRT-PCR assay was used to detect the genomic RNA of all TBEVs (nucleotide position 10,236 to 10,337). The amount of viral RNA was calculated using synthetic RNA transcript for this universal assay. Results from mouse brains were normalized using amplification (qRT-PCR) of the housekeeping gene HMBS as described previously [55]. Two specific qRT-PCR assays were also used to specifically detect either WT_IC viruses or NS5_Reenc_IC viruses (nucleotide position 8,819 to 8,933). Nucleic acids from cell supernatant media of cultured WT_IC virus or NS5_Reenc_IC virus were used as standard for these specific assays. Values for the quantity of viral RNA of each standard used for both specific assays were calculated using the universal assay.

### Tissue-culture infectious dose 50 (TCID50) assay

For each determination, a 96-well plate culture of confluent BHK21 cells was inoculated with 150μL/well of serial 10-fold dilutions of clarified (centrifugation) cell supernatant medium, mouse sera or mouse brain filtrates: each dilution was repeated 6 times. The plates were incubated for 7 days and read for absence or presence of CPE in each well. Determination of the TCID50/mL was performed using the method of Reed and Muench [56].

### ELISA test

Sera were incubated for 30min at 56°C prior to viral serology. TBEV-specific immunoglobulin G (IgG) antibodies were detected using the Anti-TBE Virus ELISA (IgG) kit (Euroimmun). Sera were diluted 1:64 and then 1:101 prior to the first incubation using the Sample Buffer of the kit. Goat anti-mouse IgG antibodies (Invitrogen) diluted 1:2000 in BSA 0.7% (KPL) as secondary antibodies were used. Plates were read using the Sunrise reader (Tecan) at a wavelength of 450nm.

### Serum neutralisation assay

Sera were incubated for 30min at 56°C prior to viral serology. For each serum, a 96-well plate culture of confluent BHK21 cells was inoculated with 50μL/well of WT_IC virus (final calculated moi: 0.001) and 50μL/well of a serial 2-fold dilution (first dilution at 1:40) of serum. Each row included 5 wells of serum dilution, a positive control (virus only) and a negative control with neither virus nor serum. The plates were incubated for 7 days and read for the absence or presence of a CPE in each well. The 50% plaque reduction neutralization titre (PRNT50/mL) was determined using the method of Reed and Muench [56].

### Statistical analysis

Kaplan-Meier survival analysis with Mandel-Cox’s Logrank tests, Student’s t tests and Fisher’s exact tests were performed using SPSS software package (IBM). *p* values below 0.05 were considered significant.

## Supporting Information

S1 Text S1–S2 Figs, S1–S4 Tables, and S1 Note. S1 Fig. Comparison between appearance of at least one symptom and weight loss.
S2 Fig. Comparative analysis of qRT-PCR and TCID50 assays.
S1 Table. Primers and probes used for the real time RT-PCR assays.
S2 Table. List of 85 TBEV sequences retrieved from GenBank.
S3 Table. List of 56 TBFV sequences retrieved from GenBank.
S4 Table. Results of weighing of challenged mice at days 11 and 12 post-challenge.
S1 Note. Re-encoded sequence (corresponding to positions 8,619–10,019 of the TBEV genome).
(PDF)
