# Supplementary material for: Attenuation of Tick-Borne Encephalitis Virus Using Large-Scale Random Codon Re-encoding
Source: PLoS Pathog. 2015 Mar 3;11(3):e1004738. doi: 10.1371/journal.ppat.1004738 (PMC4348424; doi:10.1371/journal.ppat.1004738)
Supplement: S1 Text — S1 Fig. Comparison between appearance of at least one symptom and weight loss. S2 Fig. Comparative analysis of qRT-PCR and TCID50 assays. S1 Table. Primers and probes used for the real time RT-PCR assays. S2 Table. List of 85 TBEV sequences retrieved from GenBank. S3 Table. List of 56 TBFV sequences retrieved from GenBank. S4 Table. Results of weighing of challenged mice at days 11 and 12 post-challenge. S1 Note. Re-encoded sequence (corresponding to positions 8,619–10,019 of the TBEV genome). (PDF) [file ppat.1004738.s001.pdf]

S1 Table. Primers and probes used for the real time RT-PCR assays.

System	Forward/Reverse	Sequence	Nt Position
Non specific	Forward	GCAGAGTGGGCCAGGAACAT	10,235-10,255
	Reverse	GTCATGCCGATCCATGCAGGA	10,317-10,337
	Probe	FAM-TCGGACAAGAGAAGTTCAAGGACT-TAMRA	10,288-10,311
WT specific	Forward	ACTGGCACGAAAAAGCAAACCA	8,819-8,840
	Reverse	TGCGCTTGACCACCTGTTCTG	8,913-8,933
	Probe	FAM-AGTGAAGTCCAATGCGGCTCTG-TAMRA	8,873-8,894
NS5_Reenc specific	Forward	ACTTGCCCGAAAGAGTAAGCCT	8,819-8,840
	Reverse	AGCGGATGACCATCTATTTTGC	8,912-8,933
	Probe	FAM-GGTGAAGAGTAACGCGGCTCTT-TAMRA	8,873-8,894
Inner Control	Forward	CTCTGAGAGCGGCTCTATTGGT	-
	Reverse	GTTCCCTACAACGAGCCTAAATTC	-
	Probe	FAM-TCAGACACGCGGTCCGCTATAACGA-TAMRA	-
Mouse control	Forward	TGTGTTGCACGATCCTGAAAC	-
	Reverse	CTCCTTCCAGGTGCCTCAGAA	-
	Probe	FAM-TTCGCTGCATTGCTGAAAGGG-TAMRA	-

The non-specific system detects WT-IC and NS5_Reenc_IC viruses. Specific primers and probes were designed to specifically detect either the WT_IC or NS5_Reenc_IC virus. The inner control system allowed to detect the genome of the MS2 phage (used to spike all samples). The mouse control system targets the housekeeping gene HBS and was used to normalize PCR results obtained with mouse brain samples.

S2 Table. List of 85 TBEV sequences retrieved from GenBank

GenBank accession numbers: L40361, JQ693478, AY217093, AY182009, GU183379, GU183381, GU183383, GU183384, JQ825146, JQ825161, JQ825144, JQ825158, JQ825159, JQ825164, JQ825154, JQ825156, DQ989336, JX498939, JQ825162, JQ825151, JN229223, JN003205, JF819648, EF469662, EF469661, JF316707, JF316708, JQ825155, JQ650522, JQ650523, EU816451, AF527415, DQ486861, U39292, HM120875, JN003206, AM600965, FJ402885, FJ906622, GU183382, JQ825152, JQ825160, AY169390, EU816452, EU816455, FJ997899, JQ825145, JQ825163, JQ825153, FJ402886, HQ901366, HQ901367, EU816450, HQ201303, JQ825148, JN003207, JQ825147, JQ825150, JX498940, AB062064, DQ153877, AF069066, FJ968751, EU816454, JN003208, JN003209, FJ572210, DQ401140, GQ228395, JQ825157, GQ266392, GU183380, HM535610, HM535611, HM859894, HM859895, JQ825149, EU816453, AB753012, AB062063, GU121642, JX534167, U27491, U27495, DQ862460

S3 Table . List of 56 TBFV sequences retrieved from GenBank

GenBank accession numbers: AF331718, JN860200, JF416949, JF416950, JF416951, JF416952, JF416953, JF416954, JF416955, JF416956, JF416961, JF416962, JF416963, JF416964, JF416965, JF416966, JF416967, JF416957, AF311056, DQ235145, DQ235153, DQ235146, DQ235147, AY863002, DQ462443, AY323490, EU480689, JF416959, JF416960, HM055369, JF416958, AF253419, AF253420, EU790644, Y07863, AY323489, AY193805, AY438626, AB507800, HQ231414, HM440561, HQ231415, HM440562, HM440558, HM440560, HM440559, HM440563, EU670438, EU770575, L06436, DQ235149, DQ235150, DQ235152, DQ235151, DQ235148, EU543649

S4 Table. Results of weighing of challenged mice at days 11 and 12 post-challenge.

	WT_IC				NS5_Reenc_IC			
	2.10^5 TCID50		2.10^6 TCID50		2.10^5 TCID50		2.10^6 TCID50	
	Day 11	Day 12	Day 11	Day 12	Day 11	Day 12	Day 11	Day 12
**Mouse 1**	103,6	**91,5**	105,7	101,1	104,7	106,0	97,9	98,9
**Mouse 2**	106,6	104,7	103,4	105,2	104,9	107,1	102,9	103,4
**Mouse 3**	107,8	106,3	109,3	103,4	**85,4**	94,6	104,8	105,4
**Mouse 4**	98,3	100,5	102,3	101,7	100,5	107,7	101,6	103,9

Weights are expressed as a percentage of the initial weight as describes in the Materials and Methods section.

S1 Figure. Comparison between appearance of at least one symptom and weight loss.

This figure correlates the appearance of at least one symptom appearance with the weight loss using various thresholds.

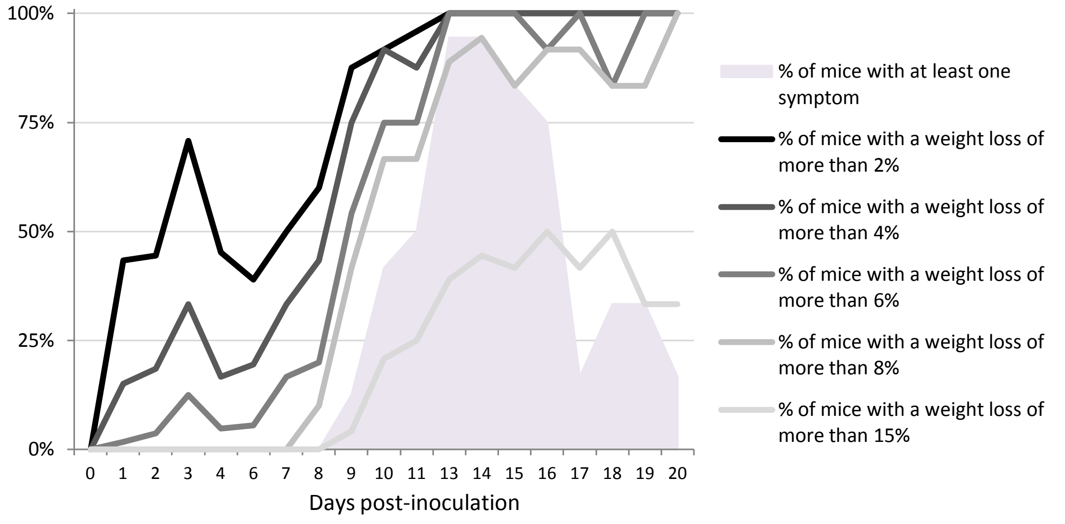

S2 Figure. Comparative analysis of qRT-PCR and TCID50 assays

Sera **(A)** and brains **(B)** collected from mice infected with the WT_IC virus (2.10^6 TCID50) were analysed using a qRT-PCR and a TCID50 assays. Samples negative with both methods were not represented in this graphic.

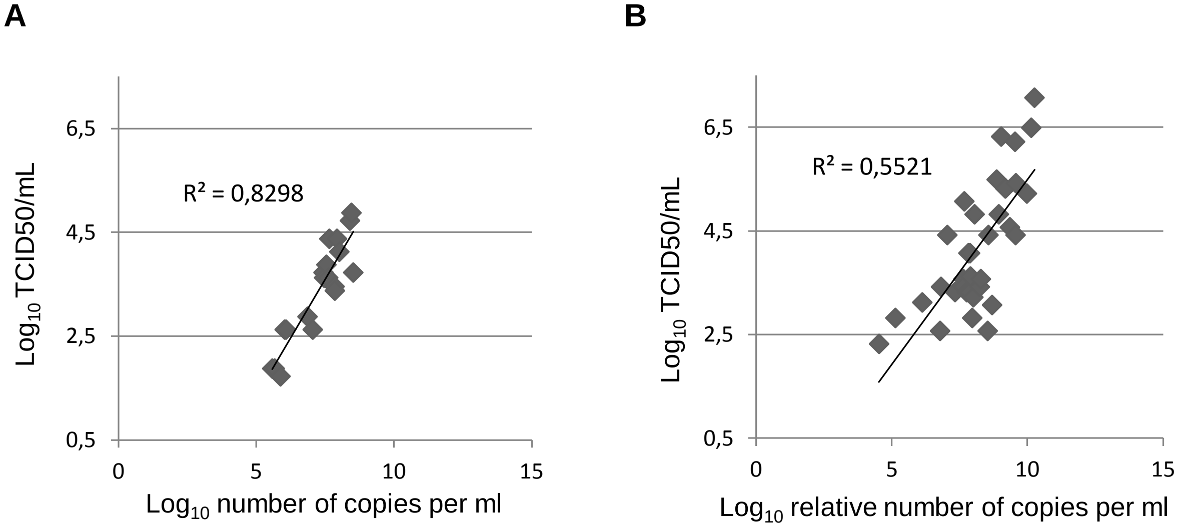

S1 Note. Re-encoded sequence (corresponding to positions 8,619 – 10,019 of the TBEV genome)

GCGAGTTTAATTAACGGAGTTGTTAAGCTCTTATCATGGCCTTGGAACGCGCGGGAGGACGTTGTCCGAATGGCAATGA
CCGATACTACCGCCTTTGGGCAGCAGCGAGTATTCAAAGAGAAGGTAGATACCAAGGCCCAGGAGCCCCAGCCAGGGA
CGAAGGTGATCATGAGGGCCGTCAATGACTGGATTCTTGAGCGACTTGCCCGAAAGAGTAAGCCTCGGATGTGTAGTAG
GGAGGAATTCATAGCGAAGGTGAAGAGTAACGCGGCTCTTGGGGCCTGGAGCGATGAGCAAAATAGATGGTCATCCGC
TAAAGAGGCCGTCGAGGACCCCGCATTTTGGCAACTGGTGGACGAGGAAAGGGAAAGACATCTGGCTGGAAGGTGCGC
ACATTGTGTCTATAACATGATGGGGAAAAGGGAGAAAAAGCTTGGAGAGTTTGGTGTTGCTAAGGGGAGTCGGGCCATT
TGGTACATGTGGCTGGGCAGCCGCTTCCTTGAGTTCGAAGCACTTGGATTTCTAAACGAGGATCACTGGGCTTCCAGGG
GGAGCTCTGGATCAGGAGTTGAAGGTATCTCCCTAAATTATTTAGGATGGTACCTAAAGGGTTTGAGCACTCTTGAGGGC
GGACTCTTTTACGCAGATGATACAGCTGGCTGGGACACTAAAGTCACAAATGCTGACCTGGAGGATGAAGAACAGCTCC
TGCGTTACATGGAAGGGGAGCATAAGCAGCTTGCGGCCACCATTATGCAGAAGGCCTATCATGCTAAGGTTGTCAAGGT
GGCACGGCCCTCCCGAGACGGTGGTTGTATAATGGATGTGATTACTAGAAGAGACCAAAGAGGCTCTGGCCAAGTAGT
GACTTATGCCCTAAACACCCTCACCAATATTAAAGTACAACTGATACGAATGATGGAAGGCGAGGGTGTCATCGAAGCAA
CGGACGCCCATAACCCAAGACTGTTTCGAGTGGAACGATGGCTCAGGGATCACGGGGAGGAACGTCTTGGGAGAATGT
TAGTTTCCGGAGATGACTGTGTAGTCAGACCTGTCGATGACAGGTTCAGTAGAGCGCTATATTTTCTGAACGATATGGCC
AAAACAAGAAAGGATGTAGGCGAGTGGGAACACTCGGTGGGTTTCTCGAATTGGGAGGAGGTTCCTTTTTGCAGTCATC
ATTTTCACGAATTAGTGATGAAAGATGGGCGCGCCTTAATAGTGCCTTGCCGAGACCAAGATGAATTGGTGGGAAGGGC
CCGCGTCTCCCCTGGGTGCGGCTGGTCAGTTCGTGAGACAGCCTGTTTGTCAAAGGCATATGGCCAAATGTGGCTTTTA
TCCTATTTTCATCGGCGCGATCTCCGAACGTTAGGTTTCGCTATCTGTTCGGCGGTCCCC
